# Antifungal effect of calcium enriched mixture cement against Candida albicans

**Published:** 2009-07-06

**Authors:** Ali Kangarlou, Samira Sofiabadi, Zahra Yadegari, Saeed Asgary

**Affiliations:** 1 *Department of Endodontics, Iranian Center for Endodontic Research, Dental School, Shahid Beheshti University of Medical Sciences, Tehran, Iran*; 2 *General Dentist, Tehran, Iran*; 3 *Researcher, Dental School, Shahid Beheshti University of Medical Sciences, Tehran, Iran*; 4 *Department, Iranian Center for Endodontic Research, Dental Research Center, Dental School, Shahid Beheshti University of Medical Sciences, Tehran, Iran*

**Keywords:** *Candida Albicans*, CEM cement, MTA, NEC, Root-end filling

## Abstract

**INTRODUCTION:** The purpose of this *ex vivo* study was to assess the effect of two root-end filling materials against *Candida (C) Albicans*.

**MATERIALS AND METHODS:** ProRoot MTA and CEM Cement were compared immediately and 24 h after mixing, in two different concentrations (50 and 100 mg/mL). A total of 50 culture wells were used and divided into experimental (n=10) and control groups (n=5). Those with no medication served as positive and without *C. Albicans *served as negative controls. All plates were incubated at 37^°^C after 1, 24, and 48hours. At each interval, the presence of *C. Albicans *was assessed and recorded by an independent observer. In addition to observing turbidity, 0.02 mL of samples from each cell was re-cultured on sabouraud dextrose agar plates to confirm change in fungal growth. The data were evaluated and analyzed using Kruskal-Wallis test.

**RESULTS:** Although all fresh and set samples with experimental concentrations showed fungal growth after 1 h; they demonstrated complete fungicidal activity at 24 and 48-h time intervals.

**CONCLUSION:** Under the conditions of this *ex vivo* study, CEM cement as well as ProRoot MTA has fungicidal effects against *C. Albicans *even in concentration of 50 mg/mL and after 24 hours.

## INTRODUCTION

Microorganisms are the main factor in creating pulpal/periapical diseases (1-2). The effects of fungus in creating endodontic diseases are also evident (3). Remnant resistant microorganisms in the root canal system or periapical tissues after primary endodontic treatment has been suggested as the main factor of root canal treatment failures (5). Interactions between bacteria in different intracanal oxygen pressures lead to colonization of specific fungal species in endodontic treatments failures (6-7). In addition, it has been shown that decrease in certain species of bacteria in root canal system may lead to excessive fungal growth with a low nutrient requirement (4). *Candida (C) Albicans* is the most common fungus of oral cavity (8). *Candida* species are not commonly present in primary root canal infections; however they are present in 20% of cases of secondary and persistent infections (9).

Contamination during endodontic treatment or recontamination as a result of coronal leakage may cause penetration of *C. Albicans* into the root canal system (3). Sustainability of *C. Albicans* in root canal system depends on its resistance against nutrient-limited environments and the action intracanal medicaments used (9). The transition of *C. Albicans* to its pathogenic form appears to be dependent on variety of virulence factors including adherence, hyphal formation, thigmotropism, protease secretion, and phenotypic switching phenomenon; these factors are capable of infecting the dentin-pulp complex (9).

Root-end resection followed by root-end filling is the treatment of choice to save a tooth when retreatment has failed or is impractical (10). The outcome depends on sufficient root-end seal and prevention of further periradicular recontamination, as well as reduction of associated bacteria/fungi. Since most of available root-end fillings do not provide a perfect and hermetic seal, their antibacterial or antifungal property is significant (11).

Mineral trioxide aggregate (MTA) was introduced for root-end fillings in 1993 (12). MTA is able to promote regeneration of adjacent periradicular tissue (13); this property, in addition to it's ability to form hydroxyapatite when contacting body tissue fluid, makes MTA a biocompatible material (14-15). MTA is not adversely affected by tissue fluid or blood contamination (16), has low cytotoxicity (17), good antibacterial effects (18), and good fungicidal effect against *C. Albicans* (19-23) as well as predictable PDL regeneration. These features make MTA a gold standard for root-end fillings. However, MTA has some disadvantages such as delayed setting time (24), poor handling characteristics (25) and high price.

Recently, a novel endodontic cement in the name of calcium enriched mixture (CEM) cement [Patent pending as Endodontic Filling Material (ENDOFILMAT)] has been introduced to dentistry (26). This material releases calcium and phosphate ions (27) and then forms hydroxyapatite not only in simulated body tissue fluid, like MTA, but also in normal saline (15). This material has similar pH to MTA, but with increased flow and decreased working time, film thickness, and estimated price than MTA (28). CEM cement stimulates hard tissue healing similar to MTA (29), form an effective seal comparable to MTA and superior to IRM; when used as root-end filling material (10,30). It also has antibacterial effect comparable to calcium hydroxide and better than MTA (11,31,32).

The purpose of this *ex vivo* study was to compare the antifungal effects of fresh and set CEM and MTA in various concentrations on *C. Albicans* at 1, 24 and 48-h time intervals.

## MATERIALS AND METHODS

The study protocol was approved by the Ethical Committee of Shahid Beheshti Medical University. In this *ex vivo* study, the two root end-filling materials ProRoot MTA (Tooth-colored, Dentsply, Tulsa Dental, Tulsa, Ok, USA) and CEM cement were prepared according to their instructions. The materials were tested in form of set and freshly mixed and also in two concentrations of 50 and 100 mg/mL.

A total of 50 culture wells were prepared for each concentration and divided into four experimental groups 1, 2, 3 and 4 (freshly mixed ProRoot MTA, freshly mixed CEM cement, 24-h set ProRoot MTA, 24-h set CEM cement respectively). Each group consisted of 10 wells each and the control groups consisted of 5 wells each.

Samples of live *C. Albicans* culture (ATCC 10231) were provided by Scientific Research Organization, Tehran, Iran and were subcultured on the surface of sabouraud dextrose agar plates. The agar plates containing the fungus were then maintained in incubator at 37^°^C. The time and condition for culture of *C. Albicans* in this study was carried out according to NCCLS standard (National Committee on Clinical Laboratory Standards) (48). The culture was diluted in a sabouraud dextrose broth media to achieve a final density of 10^4^ CFU/mL (colony forming unit/milliliter) as recommended by the NCCLS to make a suspension. One milliliter of suspension was added to the materials of each well.

Plates with 1 mL of suspension without root end-filling materials served as positive control and plates with 1 mL of suspension without *C. Albicans* served as negative control.

All study plates were incubated at 37^°^C for 1, 24, and 48 h. Growth of the *C. Albicans* was monitored daily by an independent observer; the observation was based on the presence of turbidity in the tubes.

At each time interval, 0.02 mL of samples from each well were re-cultured on sabouraud dextrose agar plates to confirm *C. Albicans* growth.

The results were evaluated and analyzed using the Kruskal-Wallis test. Statistical significance was established at P<0.05.

## RESULTS

The negative controls failed to show *C. Albicans* growth during experimented time intervals, while positive controls demonstrated fungal growth.

All culture wells associated with each experimental group showed the same results. The freshly mixed and set test materials in concentrations of 50 or 100 mg/mL did not inhibit *C. Albicans* growth after 1h.

Increasing the incubation time did not demonstrate fungal growth at 24 and 48h time intervals in all experimental groups. Microscopic evaluation of re-cultured samples of all these experimental groups confirmed that fungi have been killed in original samples. Therefore, no differences were observed among experimental groups and no statistical analysis was deemed necessary to compare the experimental groups in this respect.

## DISCUSSION

It has been recognized that the presence of *C. Albicans* is frequently associated with endodontic treatment failures (6-9). Fungicidal effect is a desirable characteristic for an ideal root-end filling material (33). Therefore, the antifungal activity of root-end filling materials has an important effect on infection control and increase of tissue regeneration. Though the ideal root-end filling material has not been discovered, MTA meets most of the properties of a perfect root-end filling. In addition to the excellent biocompatibility and chemical/ physical properties of MTA (34), the antifungal activity of this material (19-23) confirms its clinical application in endodontic surgery. MTA can therefore, be considered as the gold standard with which newer materials can be compared (*e.g. *CEM cement).

The anti-fungal/bacterial activity of root-end filling materials can be evaluated *in-vitro* by the agar diffusion test (ADT); the most commonly used method (35). However, lack of standardization of inoculum density, adequate culture medium, agar viscosity, plate storage condition, size and number of specimens per plate, and time and temperature of incubation, and reading point of the inhibition zones are factors that affect the results of ADT (36). Furthermore, when the ADT is employed for evaluating antifungal activity of materials such as MTA that have low solubility and diffusibility, the result is unreliable (23). The method used in this *ex vivo* study is the tube susceptibility test; which is an efficient method to assess antifungal properties of root-end filling materials (37). This technique allows thorough contact in the solution between *C. Albicans* and the experimental materials.

Many studies have reported the inhibitory effect of different brands of MTA on *C. Albicans* (19-23). However, several biochemical mechanisms have been proposed to explain the cytotoxicity of MTA, the antifungal action is probably related to the release of calcium hydroxide. Calcium hydroxide is produced through hydration reaction of MTA that occurs during and after mixing with water (the reactions involving tricalcium silicates and dicalcium silicates). Calcium hydroxide has been shown to eliminate bacteria by releasing hydroxyl ions, and causing an increase in pH. Alkalinity greater than 9 may reversibly or irreversibly inactivate cell membrane enzymes of the microorganisms, resulting in a loss of biological activity (38). Although many researchers reported that *C. Albicans* is highly resistant to calcium hydroxide *in-vitro *(39), others have shown that saturated solution of calcium hydroxide can effectively reduce the presence of *Candida* after a 3-min incubation (40) and completely eliminate them from bovine dentin after 7 days (41); concurring with our results.

Due to the various transitory or permanent products release after mixing, root-end filling material should be tested instantly after mixing and also after they have assumed their final chemical structure. CEM cement and MTA were tested in freshly mixed/set stages, and thus it is likely that during a period after clinical application of the material, local responses are provoked by the components of these materials with no or partial reaction. After setting, the release of active ingredients is still likely. The difference in antifungal patterns of various materials may also be related to the degree of setting (42). However, in our study, the results were similar in two different states of all tested materials.

Based on NCCLS standard 1, 24 and 48 hours time intervals were used (43). The results of our study showed that freshly mixed and set CEM cement and ProRoot MTA were effective in killing *C. Albicans* at 24 and 48 h observations, though not after 1 h. It has been shown that *C. Albicans* survived in incubation with calcium hydroxide solution for 1 and 6 hrs and was killed after 6 hrs of incubation (39), concurring with our study. Also *C. Albicans* might demonstrate resistance for only short period of time. This may explain the positive growth of the *C. Albicans* in both fresh and set materials during 1 h.

CEM cement consists of alkaline earth metal oxides/hydroxides (*e.g.* calcium oxide and calcium hydroxide), calcium phosphate and calcium silicate (10). Calcium hydroxide is present in the material itself as well as produced through the hydration reaction during and after mixing. When CEM cement is placed within the wells in contact with the medium, calcium hydroxide dissociates into Ca^2+ ^and OH^-^ and causes an increase of pH. These mechanisms may partially explain the antifungal activity of CEM cement; similar to MTA. An alternative explanation may be that the antibacterial/antifungal components of CEM cement have ample diffusion properties (11,31). The effective antifungal activity of CEM cement; comparable with MTA, indicates the good antibacterial potential of CEM cement.

Many factors characterize the biocompatibility of a root-end filling materials, such as antimicrobial/fungal activity, low cytotoxicity, genotoxicity, mutagenicity, carcinogenicity, and high histocompatibility (44). In order to evaluate these characteristics, a variety of *ex vivo* and *in vivo* studies must be carried out.

## CONCLUSION

Within the limitation of this *ex vivo* study, we can conclude that the CEM cement in concentration of 50 mg/mL has an effective antifungal activity; comparable to MTA. The promising low cost of this novel endodontic material is a significant advantage when choosing a root-end filling material. However, further *ex vivo* and *in vivo* studies are needed to assess other properties of this novel material.
